# Air pollution-derived particulate matter dysregulates hepatic Krebs cycle, glucose and lipid metabolism in mice

**DOI:** 10.1038/s41598-019-53716-y

**Published:** 2019-11-22

**Authors:** Hermes Reyes-Caballero, Xiaoquan Rao, Qiushi Sun, Marc O. Warmoes, Lin Penghui, Tom E. Sussan, Bongsoo Park, Teresa W.-M. Fan, Andrei Maiseyeu, Sanjay Rajagopalan, Geoffrey D. Girnun, Shyam Biswal

**Affiliations:** 1 Department of Environmental Health and Engineering, Johns Hopkins Bloomberg School of Public Health, 615N. Wolfe Street, Baltimore, MD 21205 USA; 2 Cardiovascular Research Institute, Case Western Reserve School of Medicine, 11100 Euclid Avenue, Cleveland, OH 44106 USA; 3 Department of Toxicology and Cancer Biology, Markey Cancer Center, Center for Environmental and Systems Biochemistry, University of Kentucky, 1095V.A. Drive, Lexington, KY 40536 USA; 4 Department of Pharmacological Sciences, Stony Brook University, BST 8-140, Stony Brook, NY 11794 USA; 5 Department of Pathology, Stony Brook University School of Medicine, Stony Brook, NY 11794 USA; 6 Present Address: Public Health Center, Toxicology Directorate, Aberdeen Proving Ground, Aberdeen, MD USA

**Keywords:** Metabolomics, Environmental impact, Type 2 diabetes

## Abstract

Exposure to ambient air particulate matter (PM_2.5_) is well established as a risk factor for cardiovascular and pulmonary disease. Both epidemiologic and controlled exposure studies in humans and animals have demonstrated an association between air pollution exposure and metabolic disorders such as diabetes. Given the central role of the liver in peripheral glucose homeostasis, we exposed mice to filtered air or PM_2.5_ for 16 weeks and examined its effect on hepatic metabolic pathways using stable isotope resolved metabolomics (SIRM) following a bolus of ^13^C_6_-glucose. Livers were analyzed for the incorporation of ^13^C into different metabolic pools by IC-FTMS or GC-MS. The relative abundance of ^13^C-glycolytic intermediates was reduced, suggesting attenuated glycolysis, a feature found in diabetes. Decreased ^13^C-Krebs cycle intermediates suggested that PM_2.5_ exposure led to a reduction in the Krebs cycle capacity. In contrast to decreased glycolysis, we observed an increase in the oxidative branch of the pentose phosphate pathway and ^13^C incorporations suggestive of enhanced capacity for the *de novo* synthesis of fatty acids. To our knowledge, this is one of the first studies to examine ^13^C_6_-glucose utilization in the liver following PM_2.5_ exposure, prior to the onset of insulin resistance (IR).

## Introduction

Exposure to ambient air particulate matter with fine (PM_2.5_) and ultrafine (PM_<2.5_) aerodynamic diameter generated from anthropogenic sources, is associated with adverse effects on human health^1^. It has been established that PM_2.5_ primarily derived from stationary and traffic-related combustion sources trigger inflammatory stress responses associated to chronic obstructive pulmonary disease (COPD), asthma and cardiovascular conditions^2–4^. Recent studies have highlighted the importance of air pollution exposure in potentiating the risk of metabolic diseases such as Type 2 Diabetes (T2D)^5^ and metabolic abnormalities^6–8^. T2D is, indeed, a major driver of cardiovascular diseases^9^ and susceptibility to T2D may represent an important but underappreciated mediator of long term risk in response to air pollution exposure. Epidemiologic studies have demonstrated association between exposure and insulin resistance (IR) and T2D^10^. In addition, controlled exposure studies have uncovered a stereotypical response to air pollution, such as excessive hepatic gluconeogenesis, fasting and post-prandial hyperglycemia, abnormalities in triglyceride lipoproteins, hepatic steatosis and inflammation^11^. In fact, we as well as other researchers have demonstrated the important role air pollution plays in non-alcoholic steatohepatitis and fatty liver dysfunction (NAFLD)^12–14^.

In this study we examined metabolic effects in response to sub-chronic (4 months) PM_2.5_ exposure by ^13^C_6_-glucose tracing in mouse livers using stable isotope resolved metabolomics (SIRM) analysis. It was of particular interest to study these effects at sub-chronic time point as opposed to chronic exposure, prior to established phenotype of insulin resistance^15^. SIRM is a recognized approach that allows analysis of metabolic network analysis based on the tracer atom labeling patterns of numerous metabolites^16^. Our results show PM_2.5_ treatment reduced glycolysis and the Krebs cycle, but enhanced the oxidative branch of the pentose phosphate pathway (OxPPP) as well as data suggestive of increased fatty acid synthesis. These metabolic changes are similar to those observed in IR and T2D; hence, may be responsible for the deleterious health effects associated to PM_2.5_ exposure and the development of metabolic disorder.

## Results

### Decreased hepatic glycolysis in PM_2.5_ exposed mice

**Figure 1:**
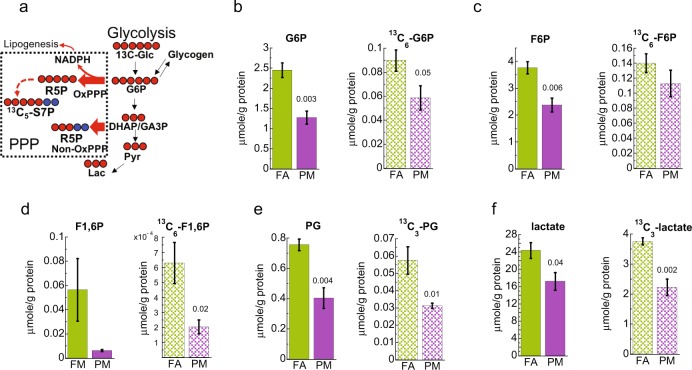
Effect of PM_2.5_ in glycolysis and pentose phosphate pathway. (**a**) Schematic of glycolysis and related pathways. PM_2.5_ exposure of mice caused a decrease in the concentration of metabolic intermediates of glycolysis. In addition, our data suggest an increase in the oxidative branch of the pentose phosphate pathway with increased production of NADPH (Fig. 3). : ^13^C; : endogenous ^12^C carbons. The width of the arrow and color code represents the magnitude of ^13^C capacity estimated according to the total accumulation of metabolites analyzed by IC-FTMS or GC-MS. (**b–f**) Glycolytic metabolites detected by IC-FTMS or GC-MS in the liver, showed an evident modulation of glucose metabolism after four months of PM_2.5_ exposure (PM) when compared to filtered air control (FA). The total shown is the average of the sum of all ^13^C and ^12^C endogenous (solid bar) and ^13^C isotopologues (textured bar). G6P, Glucose-6-phosphate; F6P, fructose-6-phosphate; F1,6P, fructose-1,6-bisphosphate; PG, 3-phosphoglycerate. Error bars represent standard error. Student *t*-test analysis of the unpaired data with equal variance (*n* = 4) and *p*-values shown deemed significant using Benjamini-Hochberg procedure (FDR = 0.1).

Previous studies show alteration of glucose utilization, including glycolysis, in the livers of animals and patients with IR or T2D^17^, and association of PM_2.5_ exposure to development of T2D and metabolic syndrome^5^. The IC-FTMS based SIRM analysis of the glucose metabolism in livers of PM_2.5_ exposed and filtered air (FA) exposed control mice produced 128 common peaks which were assigned to various metabolites related to cellular energetic pathways such as glycolysis, TCA cycle and nucleotide metabolism. Principal component analysis (PCA) of the concentration of unlabeled or total ^13^C labeled metabolites showed that samples of 16 weeks of exposure to PM_2.5_ separated from those of the control exposure to FA (Supplementary Fig. 1). PCA analysis results indicate PM_2.5_ altered the metabolism of glucose and other fuel sources in the liver. Hence, we examined the incorporation of ^13^C from uniformly ^13^C labeled glucose (^13^C_6_-Glc) into the glycolytic pathway in mice exposed to PM_2.5_ (Fig. 1a). IC-FTMS analysis exhibited a decrease in ^13^C_6_-glucose-6-phosphate, total G6P, and fructose-6-phosphate (F6P) levels in the livers of PM_2.5_ exposed mice as shown in Fig. 1b,c. Total levels of subsequent glycolytic intermediates, as well as ^13^C incorporation into fructose-1,6-bisphosphate (F1,6P), 3-phosphoglycerate (PG), and lactate also diminished based on both IC-FTMS and GC-MS analysis (Fig. 1d–f, and Supplementary Fig. 2a). These data are consistent with compromised hepatic glycolysis in PM_2.5_ compared to FA exposed mice.

**Figure 2:**
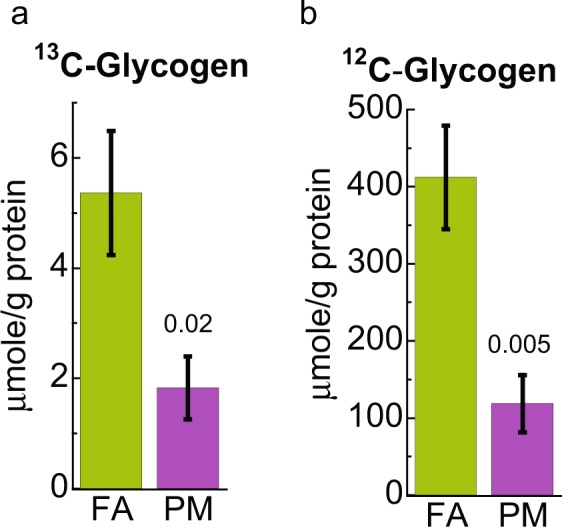
^1^H NMR analysis shows that ^13^C (**a**) and ^12^C (**b**) glycogen is reduced in the liver of mice exposed to PM_2.5_ (PM) compared to filtered air control (FA). Error bars represent standard error. Student *t*-test analysis of the unpaired data with equal variance (*n* = 5) and resulting *p*-values displayed.

It is interesting to note that the levels of both ^13^C-glycogen and unlabeled glycogen in the liver were significantly reduced in PM_2.5_ exposed mice based on NMR analysis as shown in Fig. 2a,b and Supplementary Fig. 5. The depletion of ^13^C-glycogen, together with decreased glycolysis, points to attenuated glycogen synthesis and thus decreased glycogen deposition in the liver.

### PM_2.5_ exposure increases the oxidative branch of the pentose phosphate pathway

**Figure 3:**
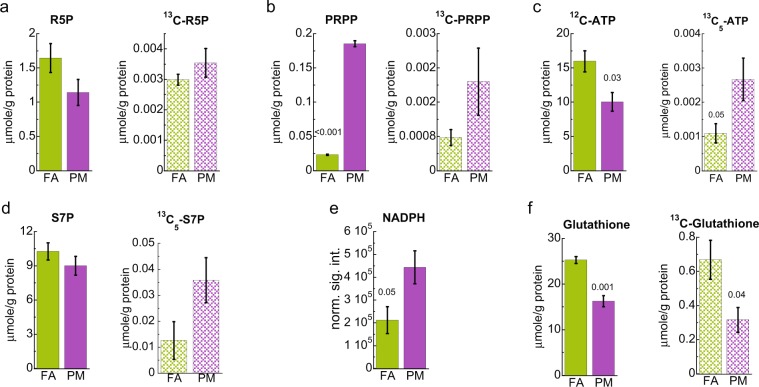
Pentose phosphate pathway (PPP) metabolites quantified by IC-FTMS. Showed is the average of the sum of all ^13^C and ^12^C isotopologues (solid bars) and sum of all ^13^C isotopologues or the specific ^13^C_*n*_ isotopologue as indicated in the text (textured bars). PM, PM_2.5_ exposed; FA, filtered air control. R5P, Ribose-5-phosphate; PRPP, phosphoribosyl pyrophosphate; S7P, sedoheptulose-7-phosphate. Error bars represent standard error. Student *t*-test analysis of the unpaired data with equal variance (*n* = 4) and *p*-values shown deemed significant using Benjamini-Hochberg procedure (FDR = 0.1).

Next, we examined ^13^C incorporation from ^13^C-glucose into PPP. IC-FTMS analysis showed a nonsignificant increase in ^13^C labeled ribose-5-phosphate (R5P) and ^13^C_5_-sedoheptulose-7-phosphate (S7P) levels along with a boost of both total and ^13^C labeled phosphoribosyl pyrophosphate (PRPP) levels in PM_2.5_ exposed mice, as depicted in Fig. 3a,b,d. Consistent with PRPP being the ribosyl donor of nucleotide synthesis, we observed a significant increase in ^13^C_5_-ATP (Fig. 3c) in PM_2.5_ exposed livers, which presumably represent ^13^C_5_-ribose in ATP^18^. Together, these results support increased carbon flow from glucose to ATP via PPP. Moreover, we noticed depletion of unlabeled (^12^C) ATP in mouse livers exposed to PM_2.5_ (Fig. 3c), which could result from decreased glycolysis and compromised Krebs cycle (see below).

The PPP consists of two arms, nonOxPPP (Fig. 1a) and oxidative (OxPPP), which produces R5P and NADPH. The substantial rise in ^13^C_5_-S7P and total NADPH levels in PM_2.5_ versus FA exposed mice livers (Fig. 3d,e; cf. Fig. 1, scheme) is consistent with enhanced OxPPP presumably in response to ROS. We also measured the incorporation of ^13^C from glucose into antioxidant glutathione (GSH) by IC-FTMS and found that both total and ^13^C labeled GSH, were reduced (Fig. 3f) in PM_2.5_ exposed mice despite NADPH accumulation. This suggests compromised GSH synthesis capacity leading to inadequate maintenance of redox homeostasis.

### Dysregulation of the hepatic Krebs cycle in PM_2.5_ exposed mice

**Figure 4:**
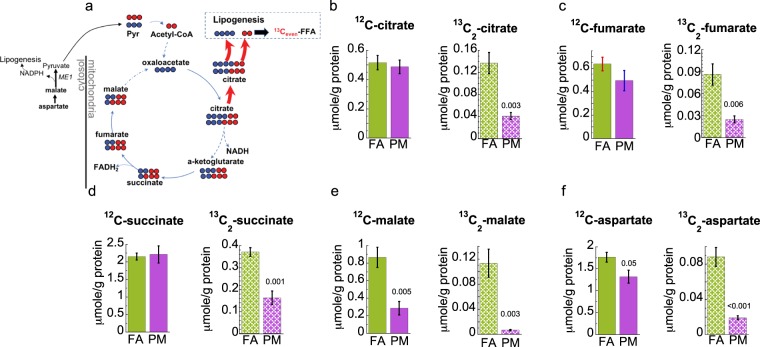
The Krebs cycle is decreased and free fatty acid (FFA) synthesis increased upon PM_2.5_ exposure. : ^13^C; : endogenous ^12^C carbons. (**a**) Schematic of the model that describes the decreased capacity of the Krebs cycle in PM_2.5_ exposed mice. The width of the arrow represents the magnitude of ^13^C flux according to the total accumulation of metabolites analyzed by IC-FTMS or GC-MS, where red arrows indicate increased and blue arrows decreased levels in PM_2.5_ exposure. Not all the isotopologues that will form are shown. (**b**–**f**) Krebs cycle metabolites analyzed by IC-FTMS or GCMS shows that a down regulation takes effect in the liver after 16 week exposure to PM_2.5_ (PM) when compared to filtered air (FA). The figure depicts unlabeled endogenous metabolite (^12^C, solid bar) and ^13^C_2_ (M + 2, dashed bars) isotopologues. For fatty acid synthesis data see Fig. 5. Error bars represent standard error. Student *t*-test analysis of the unpaired data with equal variance (*n* = 4) and *p*-values shown deemed significant using Benjamini-Hochberg procedure (FDR = 0.1).

We have previously demonstrated marked abnormalities in mitochondrial structure in response to PM_2.5_ exposure^15^. Therefore, we examined livers of PM_2.5_ and FA exposed mice for Krebs cycle intermediates and ^13^C incorporation from ^13^C_6_-glucose (Fig. 4a). Entry of ^13^C_6_-glucose derived carbons into the Krebs cycle via acetyl-CoA results in ^13^C_2_ (m + 2) labeling of citrate. ^13^C_2_-citrate was significantly reduced in PM_2.5_ exposed livers based on IC-FTMS and GC-MS analysis as shown in Fig. 4b and Supplementary Fig. 2b, respectively. We also observed reduced ^13^C_2_ labeling of Krebs cycle intermediates downstream of citrate, including succinate, fumarate and malate (Fig. 4c–e). We measured the levels of ^13^C_2_-aspartate as a surrogate metabolite for oxaloacetate (OAA), since OAA is highly labile and difficult to measure directly. ^13^C_2_-aspartate was significantly reduced in PM_2.5_ exposed livers (Fig. 4f). In addition, IC-FTMS analysis of ^13^C_3_ (m + 3) intermediates (Supplementary Fig. 3) suggests decreased entry from ^13^C_3_-pyruvate into the Krebs cycle via pyruvate carboxylation^19^. Despite the decrease in ^13^C_6_-glucose derived Krebs cycle metabolites, total unlabeled Krebs cycle intermediates were unchanged, with the exception of malate and aspartate, which decreased in PM_2.5_ liver.

### Increased hepatic lipogenesis in PM_2.5_ exposed mice

**Figure 5:**
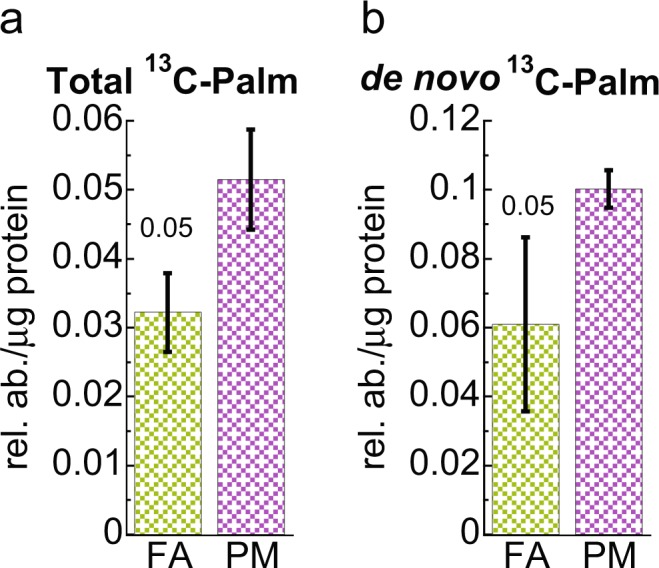
Increase palmitate in liver of mice exposed chronically to PM_2.5_. GC-MS analysis indicates that total palmitate (**a**) and *de novo* synthesis (**b**) is increased in PM_2.5_ (PM) exposed group compared to filtered air (FA). Error bars represent standard deviation. Student *t*-test analysis of the unpaired data with equal variance (*n* = 5).

One of the hallmarks of IR and T2D is the enhanced conversion of glucose into lipids^20^. The Krebs cycle-derived citrate is a precursor to fatty acid synthesis. Citrate is exported from the mitochondria by the mitochondrial citrate carrier (CIC) into the cytoplasm, where it is lysed to OAA and acetyl-CoA by the enzyme ATP citrate lyase (ACLY, see Fig. 4a). Acetyl-CoA then participates in *de novo* fatty acid synthesis with palmitate being the final product^21^. Therefore, we examined the incorporation of glucose derived ^13^C into palmitate by GC-MS. Despite the overall reduction in glycolysis and citrate synthesis, we detected increased total enrichment of ^13^C into palmitate (Fig. 5a). In addition, *de novo* fatty acid synthesis, which is reflected by enrichment of ^13^C-labeled acetyl units (derived from ^13^C glucose) into palmitate, significantly increased in PM_2.5_ exposed livers (Fig. 5b).

## Discussion

Numerous epidemiologic studies and controlled exposure studies in both humans and animal models have demonstrated that exposure to PM_2.5_ induces the metabolic syndrome^22^. Indeed, we and others had previously demonstrated obvious IR phenotype in mice exposed chronically for 10 months^15^ and sub-chronically for 10–17 weeks. However, the latter was in mice additionally receiving high fat diet, co-exposure to nickel, or some genetic background, which significantly exacerbated the phenotype^11,23,24^. Conversely, at sub-chronic exposure for 3 months or less in similar experimental conditions to this study (fasting time and diet), IR phenotype was not obvious with no change in body weight and glucose tolerance profile^24,25^. Consequently, the IR phenotype in our animal model had resulted from chronic exposure alone or sub-chronic exposure to PM_2.5_ when accompanied by other environmental or biological stressor.

We have previously demonstrated an increase in expression of PEPCK, PGC1a and defects in the hepatic insulin-AKT-FoxO1 signaling pathway resulting in overt hyperglycemia and IR in response to PM_2.5_ inhalation^11,26^. This study sought to determine if metabolic pathway abnormalities in the liver precede the development of overt IR and T2D by examining changes in metabolites of intermediary metabolism from the livers of mice exposed to PM_2.5_ prior to the development of IR and T2D (Supplementary Fig. 4). Hence, we performed ^13^C stable isotope tracer approach to determine how PM_2.5_ exposure alters glucose utilization in the liver prior to IR and T2D development. We showed that PM_2.5_ exposure leads to changes in hepatic metabolism associated with T2D and IR, even before detecting evidence of their manifestation. Previous studies in cardiac tissues from mice exposed to acute PM_2.5_ showed reduced glycolysis^27^. In agreement, we observed depletion of ^13^C-labeled and total (^12^C + ^13^C) glycolytic products including lactate from the livers of mice exposed to PM_2.5_. Our data suggests that exposure to PM_2.5_ results in a decreased hepatic glycolytic activity derived from glucose and non-glucose precursors like glycogen.

^13^C incorporation into Krebs cycle intermediates were also reduced, signifying compromised Krebs cycle capacity. Previous studies exhibit increased anaplerotic input of non-carbohydrate substrates such as propionate, lactate or glucogenic amino acids into the Krebs cycle in diabetic rodents and humans^28^. This led to increased gluconeogenesis via the pyruvate carboxylase/PEPCK pathway and increased hepatic glucose output. Thus, it is not surprising that diabetes is associated to reduce oxidation of glucose via the Krebs cycle^29,30^. Our data supports that even prior to hyperglycemia, there was a reduction in glucose oxidation while anaplerosis was also attenuated based on m + 2 and m + 3 labeling of the Krebs cycle products. Furthermore, the majority of unlabeled Krebs cycle products did not change (Fig. 4), which could result from the replenishment by unlabeled substrates such as amino acids, other non-carbohydrate sources, and glycogen in metabolism adapted to prolonged fast^31^. A common finding in previous reports is increased accumulation of amino acids in the blood and the lungs, and dysregulation of amino acids in the liver, testis and heart after exposure to PM_2.5_^27,32–36^. In the context of unlabeled sources of carbons, the data supports accelerated use of endogenous aspartate and malate in response to PM_2.5_ for PEP production by PEPCK as previously observed during decreased availability of glycolytic precursors^37,38^ and as is suggested by previously reported increased aspartate utilization in the lungs after acute PM_2.5_ exposure^34^. In our study, it seems plausible that dysfunctional glucose metabolism is balanced out by use of alternative fuels. Of note is the reduced glycogen synthesis from glucose in mice expose to PM_2.5_ (Fig. 2) which is characteristic of IR in agreement with our previous studies^8^. The depletion of unlabeled glycogen suggests reduced synthesis from sources other than glucose and/or enhanced glycogen catabolism (glycogenolysis) in PM_2.5_ exposed mice, either of which could increases glucose disposal into the circulation^39,40^. Moreover, reduced glycogen synthesis may be linked to increased fatty acid synthesis^41^. Accumulation of palmitate may inhibit glycogen production while mediating hepatic insulin resistant phenotype^42^.

Nevertheless, despite the reduced levels of the ^13^C-citrate precursor, incorporation of ^13^C into fatty acids was enhanced. This suggests that ^13^C-citrate was depleted in part due to its enhanced use in *de novo* fatty acid synthesis. Our results on fatty acid synthesis are in agreement with previously reported systemic imbalance of lipid homeostasis after exposure to PM_10_^43^, in NAFLD^44^, and increased blood lipids after chronic exposure to PM_2.5_^36^. Indeed, at a late stage of progression to NASH or T2D, the mitochondrial respiration, ATP synthesis and rate of lipid oxidation in the liver is compromised^45,46^, which can impair insulin signaling and increase inflammation^47,48^. How this changes occur is ukknonwn, nevertheless, we presented evidence that a metabolic effect of PM_2.5_ exposure is compromised oxidation of glucose compensated by the use of alternative fuels. In line with our results, reported effects of acute PM_2.5_ exposure are decreased ATP in cardiomyocytes, decreased of fumarate accumulation in the liver, and the lungs^27,33,34,49^. However, we recognized that our assessment is limited to the liver metabolism of a single time point, that is insufficient to describe without ambiguity the kinetics and directionality of the metabolic reactions.

Interestingly, an increase in both branches (OxPPP and nonOxPPP) of the PPP is likely a consequence of the PM_2.5_ exposure. OxPPP produces R5P and NADPH, whereas nonOxPPP is reversible and switches between R5P generation from glycolytic intermediates or generation of glycolytic intermediates from R5P coming from OxPPP. This allows cells to alternate between R5P or NADPH and R5P production. Thus, OxPPP could be the predominant PPP when NADPH is needed for biosynthetic reactions and in response to oxidative stress. Therefore, increased NADPH accumulation suggests increased OxPPP, although we cannot rule out changes to other sources of NADPH. For example, recent reports suggest that malic enzyme (ME1) supplied NADPH for lipogenesis in prediabetic liver^50^. The requirement of NADPH by fatty acid synthesis would reduce NADPH availability for redox homeostasis. In line with NADPH clearance by reductive biosynthesis, we observed decreased *de novo* synthesis and total GSH. Particulate matter shows oxidative potential, and many studies note that exposure to the constituents of PM_2.5_ or PM_0.1_ has deleterious effects on the liver via reactive oxygen species (ROS) production^51^; black carbon nanoparticles^52,53^, graphene oxide^54^, TiO nanoparticles^55^, nickel^56^ and even the PM_2.5_ mixture^57,58^, have been shown to promote liver damage via ROS. In addition, previous studies showed that acute exposure to PM_2.5_ increased ROS in cells and decreased levels of GSH in the liver with similar results obtained for chronic exposure^33,59,60^. Therefore, our studies support compromised GSH synthesis as potentially an additional mechanism for the increase in oxidative stress following chronic exposure to PM_2.5_.

The mice exposed to PM_2.5_ showed no differences relative to filtered air exposed mice with regard to weight change or glucose tolerance at this early time point (Supplementary Fig. 4). Therefore, we assert to show that metabolic changes precede IR/diabetes onset in PM_2.5_ exposed mice. Indeed, previously published results showed redox imbalance in kidney of mice chronically exposed to PM_2.5_ and a similar metabolic phenotype after acute exposure in cardiac tissue and hepatic cells that the one reported here for the liver^27,61,62^. We acknowledge a limitation in our study is that molecular bases of metabolic dysregulation cannot be identified, a subject matter of future research. However, our results suggest an insight into the mechanism; the early responses of attenuated glycolysis, the Krebs cycle, and GSH synthesis, as well as increased lipogenesis, induce an imbalance of reductive capacity in the liver, leading to disrupted redox homeostasis in the liver of PM_2.5_ exposed mice.The dysrupt redox homeostasis could in turn lead to inflammation, increased fibrosis, and lipid steatosis in liver due to increased ROS and the subsequent development of IR and T2D^12,63–66^.

## Materials and Methods

### Animals

Seven-week-old male C57BL/6J mice were purchased from Jackson Laboratories (Bar Harbor, ME) and were equilibrated for 2 weeks before exposure. All mice were housed in cages with normal chow diet at 21 °C on a 12-h. light/12-h. dark cycle with free access to water and food. The protocols and the use of animals were approved by and in accordance with Animal Care and Use Committee (IACUC) at University of Maryland (protocol number 1113011) and Johns Hopkins University (protocol number M013H134), Baltimore. The performance of all experiments followed the recommendations and guidelines from IACUC, including all the methodlogy and procedures relevant to mice experimentation in this research.

### Ambient whole-body inhalation protocol

C57BL/6J mice were exposed to filtered air (FA) or concentrated ambient PM_2.5_ in a mobile trailer located on the campus of the University of Maryland, Baltimore. The concentrated PM_2.5_ was generated using a versatile aerosol concentration enrichment system (VACES) as described previously^67^. Mice were exposed to concentrated PM_2.5_ particles for 6 hours per day, 5 days per week for a total of 16 weeks. The control group (mice exposed to FA) in the experiment were exposed to an identical protocol with all PM_2.5_ particles removed by a high-efficiency particulate-air filter positioned in the inlet valve. The average concentrations of PM_2.5_ during the exposure period were 8.7 ± 2.6 μg/m^3^ in the ambient air and 62.5 ± 21.3 μg/m^3^ in the concentrated PM chamber. The mean PM_2.5_ levels (±SD) in the current study (62.5 ± 21.3 μg/m^3^) were similar within standard deviation to our prior report (69.6 ± 48.4 μg/m^3^) of sub-chronic 3-month exposure and co-exposure with nickel (Ni)^24^. The chemical composition of the PM_2.5_ mixture in this study is unavialable, however a mixture in a near by site was characterized two years ealier^68^.

### Intraperitoneal glucose tolerance test protocol

A week before the end of the exposure period we performed a previously reported intraperitoneal glucose tolerance test (IPGTT) procedure^15^. Glucose tolerance test required mice fasted for 12 hours (including the mice we did the ^13^C-glucose injection), which results in normal glucose use in the liver^69^. Briefley, mice were weighted and then injected intraperitoneally with glucose (2 mg/kg body weight). Blood samples were collected through the tail vein and glucose concentrations were measured before and 30, 60, 90, and 120 min after the injection on an Elite Glucometer (Bayer, Leverkusen, Germany). Supplementary Fig. 5 reports body weights, IPGTT plot, and area under the curve calculated using GraphPad software with standard error computed (*n* = 10).

### ^13^C_6_-glucose administration and tissue harvest

On the following day of the final exposure, mice were injected intraperitoneally with ^13^C_6_-glucose (Cambridge Isotopes Laboratories) according to the protocol described by Fan *et al*. with modification^70^. 80 μl (20 mg) of ^13^C_6_-glucose in PBS were injected at 15 min. intervals for three times to mice fasted for 12 hours. Time of injection was recorded and blood was collected before and after injection. Liver tissues were collected at 45 min. after the first injection (15 min. after the last injection) and frozen immediately (within 5 min. of necropsy) in liquid N_2_.

### Sample preparation

Quenching and extraction of liver samples (left lobules) were performed as previously described^71^. Briefly, tissues were pulverized into <10 µm powder in liquid nitrogen using a Spex Freezer Mill (SPEX SamplePrep, Metuchen, NJ, USA). Metabolites and proteins were then extracted in a final 2:1.5:1 ratio of acetonitrile:H_2_O:chloroform. Protein content was determined using the Pierce BCA method (Thermo Fisher Scientific, Rockford, IL) for normalizing the metabolite concentration.

The polar fractions were aliquoted and lyophilized for NMR and IC-FTMS analysis. The NMR fractions were further deproteinated in 80% acetone solution (100 µL ice-cold nanopure water and 400 µL ice-cold 100% acetone), incubated at −80 °C for 30 min., followed by centrifugation at 4 °C, 14,000 rpm for 20 min. The supernatant was lyophilized.

### Gas chromatography-mass spectrometry

Polar and non-polar (for fatty acids) fractions were isolated as previously described^72–74^. In brief, livers were homogenized in 0.9% NaCl, centrifuged and a modified Folch extraction performed using 2:1:0.2 0.9% chloroform:methanol:NaCl for non-polar metabolites. For polar metabolites, livers were homogenized in 80% methanol, freezed thawed 3 times, centrifuged and supernatant collected. Fatty acids were saponified and converted to their methylated derivatives. Palmitate and its isotopomers were monitored at *m/z* 270–286 by gas chromatography-mass spectrometry (GC-MS). The enrichment of acetyl-CoA units and *de novo* palmitate synthesis were determined as previously described^73,74^. Briefly, for acetyl CoA enrichment by the formula of m4/m/2 = (n-1)/2(p•q), where n equals the number of acetyl units, for palmitate = 8; p is the ^13^C labeled precursor acetate fraction and q is the ^12^C acetate fraction. For polar metabolites samples were methoximated, derivatized using BSTFA and analyzed by GCMS^74^. The m/z for the following metabolites and their isotopologues were monitored: lactate 219–222 m/z, m + 3 = 222; 3PG 459–462 m/z; citrate 465–471 m/z, m + 2 = 467; fumarate m/z = 245–249, m + 2 = 247; succinate m/z = 247–251, m + 2 = 249; Malate m/z = 335–339, m + 2 = 337; Aspartate m/z = 334–338, m + 2 = 336, 3PG m/z = 459–462, m + 3 = 462. Data were analyzed using Mass Hunter (Agilent, USA) and abundance corrected using ISOCOR.

### Nuclear magnetic resonance (NMR)

1D ^1^H and ^1^H (^13^C) HSQC (heteronuclear single quantum coherence) NMR analyses of polar extracts reconstituted in D_2_O (>99.9%, Cambridge Isotope Laboratories, MA) containing 0.1 mM EDTA (Ethylenediaminetetraacetic acid, Sigma Aldrich, St. Louis, MO) and 0.5 mM d6-2,2-dimethyl-2-silapentane-5-sulfonate (DSS) (Cambridge Isotope Laboratories, Tewksbury, MA) as internal standard were performed on a DD2 14.1 Tesla NMR spectrometer (Agilent Technologies, CA) equipped with a 3 mm inverse triple resonance HCN cryoprobe. 1D ^1^H spectra were acquired with standard PRESAT pulse sequence at 15 °C. A total of 16384 data points were acquired with 2 s. acquisition time, 512 transients, 12 ppm spectral width, and 4 s. recycle delay time. The spectra were then linear predicted and zero filled to 128k points and apodized with 1 Hz exponential line broadening. 1D HSQC spectra were recorded with ^13^C adiabatic decoupling scheme for broad range decoupling during proton acquisition time of 0.25 s. 1796 data points were collected for each transient and a total of 1024 transients were acquired with 12 ppm spectral width. The HSQC spectra were then apodized with unshifted Gaussian function and 4 Hz exponential line broadening and zero-filled to 16k data points before Fourier transformation. Metabolites were assigned by comparison with in-house^75^ and public NMR databases. Metabolite and their ^13^C isotopomers were quantified using the MestReNova software (Mestrelab, Spain) by peak deconvolution. The peak intensities of metabolites obtained were converted into nmoles by calibration against the peak intensity of DSS (27.5 nmoles) at 0 ppm for 1 H spectra. For HSQC spectra, ^13^C-3-lactate was quantified by the two ^13^C satellite peaks in the 1D ^1^H spectra, which was used as the internal calibration standard for quantifying other assigned HSQC signals, as described previously^76^. Quantified metabolites were normalized to total protein weight for each sample.

### Ion chromatography-fourier transform mass spectrometry (IC-FTMS)

Polar extracts for IC-FTMS were reconstituted in 20 µL ultrapure water (EMD Millipore) of which 10 µL was used for IC-FTMS. All IC-FTMS analyses were performed on a Dionex ICS-5000+ ion chromatography interfaced to a Thermo Fusion Orbitrap Tribrid mass spectrometer (Thermo Fisher Scientific) as previously described^77^, with the exception that we used a scan range of 80 to 700 m/z. Isotopologue peak areas were integrated and exported to Excel via the Thermo TraceFinder (version 3.3) software package. Natural abundance correction of peak areas was performed as described previously^78^. Fractional enrichment was calculated as the percentage of the natural abundance corrected signal of each isotopologue from the sum of all isotopologues for given metabolite and averaged across all replicates. Quantification for selected metabolites was achieved by using an external standard mixture and normalization to protein weight. Principal component analysis (PCA) used the *pca* function of the MATLB software version R2018a. The statistical parameters of PCAs are in Supplementary Tables (1 and 2). Pair way comparation analysis used the Benjamin-Hochberg correction for multiple testing and the Student t-test to calculate *p-*values of ^12^C and ^13^C-metabolites analyzed by IC-FTMS as shown in the figures (FDR ≤ 0.1).

## Supplementary information

Supplementary Information

## Data Availability

The data that support the findings of this study are available upon request to anyone with a public interest. Please send and email to the corresponding author where you clearly indicate your affiliations.
