# Author Correction: Air pollution-derived particulate matter dysregulates hepatic Krebs cycle, glucose and lipid metabolism in mice

**DOI:** 10.1038/s41598-020-60083-6

**Published:** 2020-03-16

**Authors:** Hermes Reyes-caballero, Xiaoquan Rao, Qiushi Sun, Marc O. Warmoes, Penghui Lin, Tom E. Sussan, Bongsoo Park, Teresa W.-M. Fan, Andrei Maiseyeu, Sanjay Rajagopalan, Geoffrey D. Girnun, Shyam Biswal

**Affiliations:** 1 Department of Environmental Health and Engineering, Johns Hopkins Bloomberg School of Public Health, 615N. Wolfe Street, Baltimore, MD 21205 USA; 2 Cardiovascular Research Institute, Case Western Reserve School of Medicine, 11100 Euclid Avenue, Cleveland, OH 44106 USA; 3 Department of Toxicology and Cancer Biology, Markey Cancer Center, Center for Environmental and Systems Biochemistry, University of Kentucky, 1095V.A. Drive, Lexington, KY 40536 USA; 4 Department of Pharmacological Sciences, Stony Brook University, BST 8-140, Stony Brook, NY 11794 USA; 5 Department of Pathology, Stony Brook University School of Medicine, Stony Brook, NY 11794 USA; 6 Present Address: Public Health Center, Toxicology Directorate, Aberdeen Proving Ground, Aberdeen, MD USA

Correction to: *Scientific Reports* 10.1038/s41598-019-53716-y, published online 22 November 2019

The original version of this Article contained an error in the name of the author Penghui Lin which was incorrectly given as Lin Penghui.

This error has now been corrected in the PDF and HTML versions of the Article, and in the accompanying Supplementary Information file.

