# Supplementary material for: Air pollution-derived particulate matter dysregulates hepatic Krebs cycle, glucose and lipid metabolism in mice
Source: Sci Rep. 2019 Nov 22;9:17423. doi: 10.1038/s41598-019-53716-y (PMC6874681; doi:10.1038/s41598-019-53716-y)
Supplement: Supplementary file 1 — Supplementary Information [file 41598_2019_53716_MOESM1_ESM.pdf]

Supplementary Figures

Air pollution-derived particulate matter dysregulates hepatic Krebs cycle, glucose and lipid metabolism in mice.

Hermes Reyes-Caballero[1,*], Xiaoquan Rao[2], Qiushi Sun[3], Marc O Warmoes[3], Lin Penghui[3], Tom E. Sussan[1,#], Bongsoo Park[1], Teresa W.-M. Fan[3], Andrei Maiseyeu[2], Sanjay Rajagopalan[2], Geoffrey D Girnun[4], Shyam Biswal[1*]

[1]Department of Environmental Health and Engineering, Johns Hopkins Bloomberg School of Public Health, 615 N. Wolfe Street, Baltimore, MD 21205, USA.

[2] Cardiovascular Research Institute, Case Western Reserve School of Medicine, 11100 Euclid Avenue, Cleveland, OH 44106, USA.

[3]Department of Toxicology and Cancer Biology, Markey Cancer Center, Center for Environmental and Systems Biochemistry, University of Kentucky, 1095 V.A. Drive, Lexington, KY 40536, USA.

[4]Department of Pharmacological Sciences, Stony Brook University, Stony Brook, BST 8-140, NY 11794, USA; Department of Pathology, Stony Brook University School of Medicine, Stony Brook, NY 11794, USA.

[#]Current affiliation: Public Health Center, Toxicology Directorate, Aberdeen Proving Ground, MD

*Correspondence should be addressed to: hreyesc1@jhu.edu; Sbiswal@jhu.edu

Supplementary Figure 1

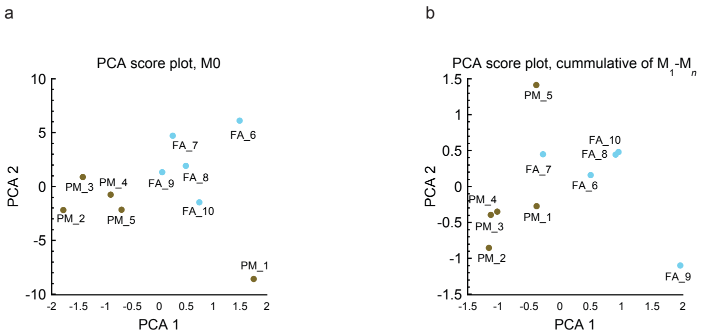

Supplementary Figure 1 PCA data reduction of liver metabolites from $PM_{2.5}$ and filter air exposed mice. Plane projection of the two first principal components for variance explanation of metabolites quantified using IC-FTMS analysis. The computation included the intensity under the area of 128 signals (variables) after data was normalized to protein concentration in 5 liver samples from each group. Outliers were identified as PCs values that fall outside the interquartile range in each group. •: filtered air (FA), •: $PM_{2.5}$ exposed (PM). (a) All unlabeled metabolites (M0). 80% and 90% of variance is explaned by PC1 and PC2, respectively. Samples 1 and 6 are outliers and excluded from further analysis. (b) PCA analysis of the sum of all ^{13}C isotopologues ($\Sigma^{13}C_1 \ldots {}^{13}C_i$). 58% and 30% of variance is explaned by PC1 and PC2, respectively. Samples 5 and 9 are outliers and were excluded from further analysis. Statistics of PCA are in Suplementary Table(1,2).

Supplementary Figure 2

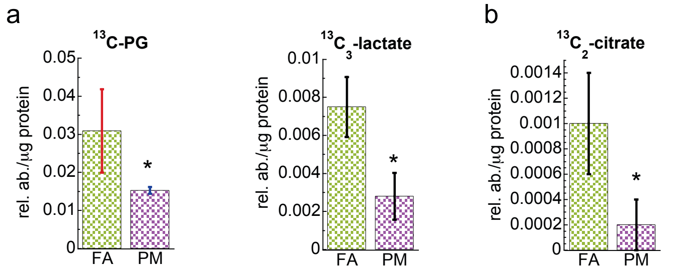

Supplementary Figure 2 Metabolites detected by GC-MS in liver. $PM_{2.5}$ exposure (PM) and filter air control (FA). (a) Glycolysis metabolites. Total showed is the total ^{13}C-PG (3-phosphoglycerate) and the $^{13}C_3$-lactate isotopologue. (b) Krebs cycle metabolite $^{13}C_2$-citrate. Error bars represent standard error. Student t-test analysis of the unpaired data with equal variance (n=4): $p \leq 0.05$ (*).

Supplementary Figure 3

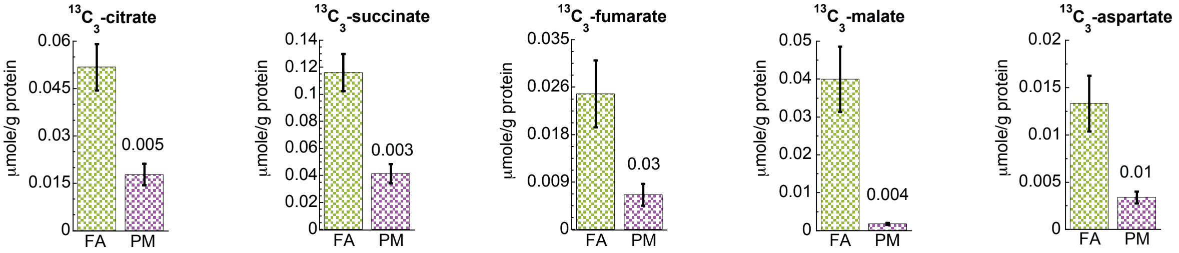

Supplementary Figure 3 Krebs cycle metabolites analyzed by IC-FTMS show that a down regulation takes effect in liver after 16 weeks exposure of mice to $PM_{2.5}$ (PM) compared to filtered air control (FA). The figure depicts $^{13}C_3$ isotopologues. Error bars are standard error. Student *t*-test, unpaired data with equal variance (n=4) and p-values shown deemed significant using Benjamini-Hochberg procedure (FDR = 0.1).

Supplementary Figure 4

a b c

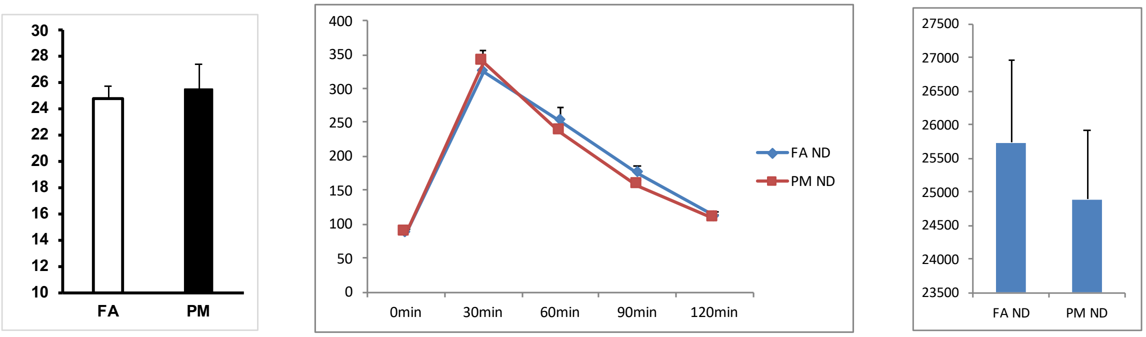

Supplementary Figure 4 Glucose tolerance and body weights unchanged after $PM_{2.5}$ exposure. (a) Body weights of the mice before organ collection. Error bars represent standard deviation. (b) Intraperitoneal glucose tolerance test (IPGTT) and (c) area under the curve (AUC). PM, $PM_{2.5}$ exposed; FA, filtered air control.

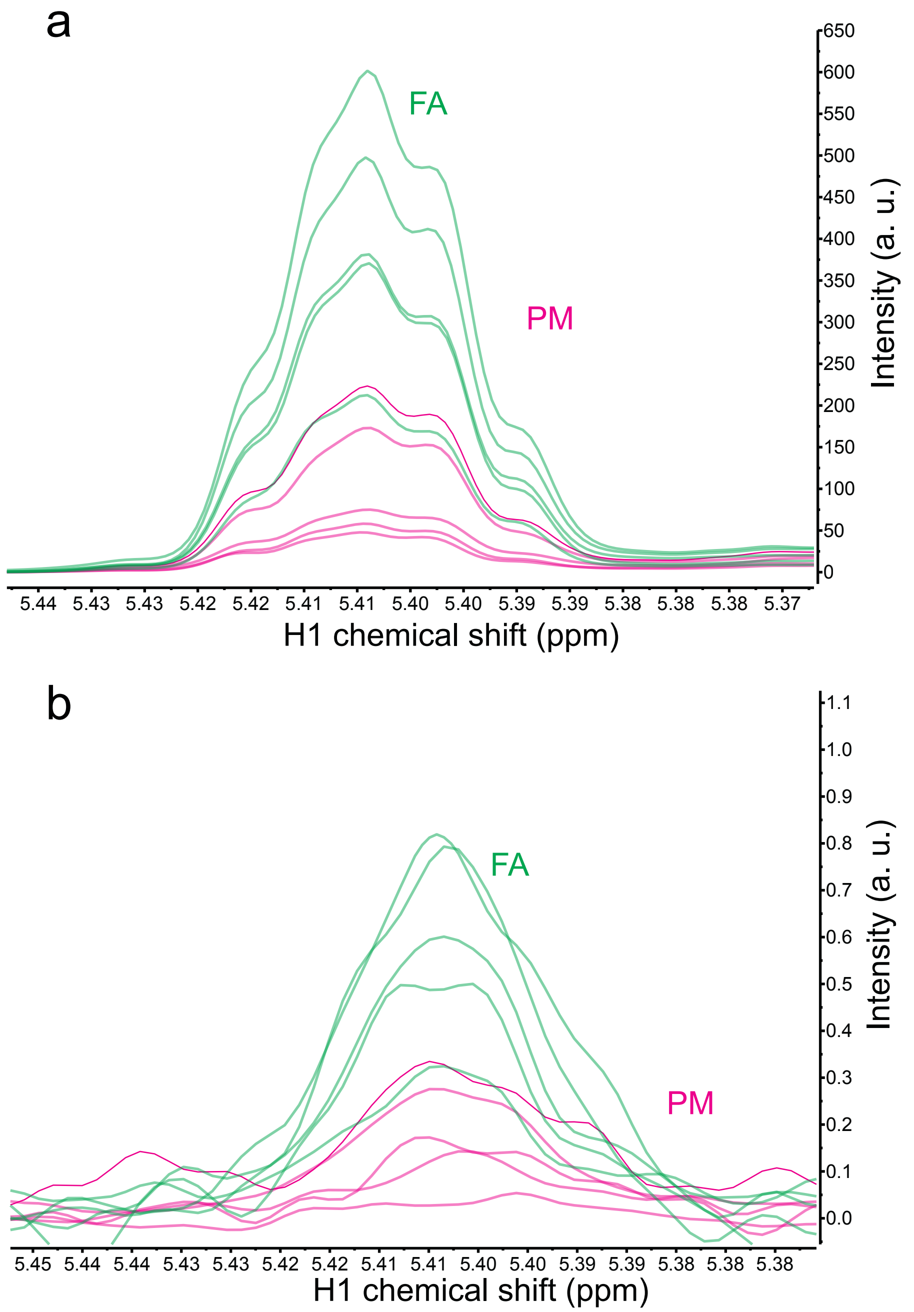

Supplementary Figure 5 Glycogen extracted from the liver analyzed by nuclear magnetic resonance (NMR). 1D 1H (a) and 1H (13C) HSQC (heteronuclear single quantum coherence) spectral NMR region corresponding to glycogen (b). The Human Metabolome Database (HMDB) entry 0000757 facilitated peak assignment. Shown is superimposed spectra normalized to protein. Spectrum lines colored green: mice exposed to filtered air (FA), and magenta: mice exposed to PM2.5 (PM). a. u: arbitrary units.

Supplementary Tables

Air pollution-derived particulate matter dysregulates hepatic Krebs cycle, glucose and lipid metabolism in mice.

Hermes Reyes-Caballero[1,*], Xiaoquan Rao[2], Qiushi Sun[3], Marc O Warmoes[3], Lin Penghui[3], Tom E. Sussan[1,#], Bongsoo Park[1], Teresa W.-M. Fan[3], Andrei Maiseyeu[2], Sanjay Rajagopalan[2], Geoffrey D Girnun[4], Shyam Biswal[1*]

[1]Department of Environmental Health and Engineering, Johns Hopkins Bloomberg School of Public Health, 615 N. Wolfe Street, Baltimore, MD 21205, USA.

[2] Cardiovascular Research Institute, Case Western Reserve School of Medicine, 11100 Euclid Avenue, Cleveland, OH 44106, USA.

[3]Department of Toxicology and Cancer Biology, Markey Cancer Center, Center for Environmental and Systems Biochemistry, University of Kentucky, 1095 V.A. Drive, Lexington, KY 40536, USA.

[4]Department of Pharmacological Sciences, Stony Brook University, Stony Brook, BST 8-140, NY 11794, USA; Department of Pathology, Stony Brook University School of Medicine, Stony Brook, NY 11794, USA.

[#]Current affiliation: Public Health Center, Toxicology Directorate, Aberdeen Proving Ground, MD

*Correspondence should be addressed to: hreyesc1@jhu.edu; Sbiswal@jhu.edu

Supplementary Tables

Supplementary Table 1 Eigenvalues and the fraction of the variation that is explained by the principal components analysis of metabolites

PC	Eigenvalue	±SD	Proportion	Cumulative
PCA of ^{13}C isotopologues ($\sum {}^{13}C_1 \ldots {}^{13}C_i$)				
1	1.09E+14	1.05E+07	0.58	0.58
2	5.47E+13	7.40E+06	0.29	0.88
3	1.45E+13	3.80E+06	0.08	0.95
PCA of all unlabeled metabolites (M_0)				
1	1.41E+16	1.19E+08	0.804	0.804
2	1.68E+15	4.10E+07	0.096	0.900
3	8.36E+14	2.89E+07	0.048	0.947

Supplementary Table 2. Metabolites with the highest correlation to the PC1 and PC2 coefficients (PCA loadings).

**Metabolite**	**PC1**	**PC2**
PCA of all ^{13}C isotopologues ($\sum {}^{13}C_1 \ldots {}^{13}C_i$)		
GSH	**0.732**	**-0.422**
Succinate	**0.313**	0.144
Lactate	**0.281**	**0.704**
Citrate	**0.210**	0.050
Malate	**0.154**	0.089
Glycerol-3-phosphate	**0.080**	**0.455**
PCA of all unlabeled metabolites (M_0)		
GSH	**0.889**	**-0.233**
Glycerol-3-phosphate	**0.165**	**0.374**
ATP	**0.124**	**0.584**
F6P	**0.045**	**0.086**
